# Neutrophil phenotype model for extracorporeal treatment of sepsis

**DOI:** 10.1186/cc13423

**Published:** 2014-03-17

**Authors:** A Malkin, R Sheehan, S Mathew, H Redl, G Clermont

**Affiliations:** 1 University of Pittsburgh, PA, USA; 2 Ludwig Boltzmann Institute for Experimental and Clinical Traumatology, Vienna, Austria

## Introduction

Neutrophils play a central role in eliminating bacterial pathogens, but may also contribute to end-organ damage. Interleukin-8 (IL-8), a key modulator of neutrophil function, signals through neutrophil specific surface receptors CXCR-1 and CXCR-2. Expression of these surface receptors can be altered by perfusion through an extracorporeal device. Extracorporeal methods of immune modulation have shown promise for treatment of sepsis; however, achieving an appropriate response is a major challenge [1]. In this study a mechanistic mathematical model was used to evaluate and deploy an extracorporeal sepsis treatment that modulates CXCR-1/2 levels.

## Methods

A simplified mechanistic mathematical model of IL-8- mediated activation of CXCR-1/2 receptors was developed. Receptor-level dynamics and systemic parameters are coupled with multiple neutrophil phenotypes to generate dynamic populations of activated neutrophils that reduce pathogen load, and/or primed neutrophils that cause adverse tissue damage when misdirected. The mathematical model was calibrated using experimental data from baboons administered a 2-hour infusion of E. coli and followed for a maximum of 28 days. An extracorporeal intervention was implemented by introducing a trapped receptor state that limits IL-8 signaling through CXCR-1/2. Time of onset, duration, and capture efficacy of the extracorporeal device were explored to provide probabilistic predictions of the impact on mortality.

## Results

Of 16 baboons, 11 (69%) died, six (38%) within 1 day of bacterial infusion. The model was well calibrated to the data from survivors and nonsurvivors. Sensitivity analysis identified six model parameters, out of 21, as key determinants of mortality. Predictions indicate best effect with introduction of the proposed extracorporeal intervention within 1 hour of infection for a 72-hour duration, results in the survivor population increasing from 31% to 61%. The treatment can result in harm if initiated <10 hours from infection and continued <24 hours. A delay of 10 hours increases survival by 7% on average. Treatment efficacy quickly diminishes if not introduced within 15 hours of infection.

## Conclusion

These findings support the continued development of an extracorporeal treatment that modulates CXCR-1/2 levels. Further development of the mathematical model will help guide device development and determine which patient populations should be targeted by treatment.
